# Neuroendocrine and neural control of bone mass in health and disease

**DOI:** 10.1172/JCI203623

**Published:** 2026-05-01

**Authors:** Mone Zaidi, Se-Min Kim, Vitaly Ryu, Daria Lizneva, Terry F. Davies, Clifford J. Rosen, Tony Yuen, Andrea Giustina

**Affiliations:** 1 Institute for Translational Medicine and Pharmacology, Icahn School of Medicine, New York, New York, USA.; 2 Maine Health Institute for Research, Scarborough, Maine, USA.; 3 Institute of Endocrine and Metabolic Sciences, IRCCS San Raffaele Hospital and San Raffaele Vita-Salute University, Milan, Italy.

## Abstract

Bone is a highly dynamic and purposefully organized structure that remodels constantly throughout adult life. Disordered bone remodeling, in which resorption of old bone by osteoclasts exceeds new bone formation by osteoblasts, results in bone loss, which, in turn, is associated with debilitating conditions, including osteoporosis and metastatic bone disease. The past decade has revealed vital new insights into the role of the central nervous system in skeletal regulation. These studies have led to a better understanding of physiologic circuitry, enabled us to revisit disease pathophysiology, and in doing so, prompted the creation of candidate therapeutics. The central neural control of bone is exerted through two arms — an amplitude-modulated (AM) neurohormonal arm that relies on changes in circulating levels of anterior and posterior pituitary hormones, which act on bone directly, and a frequency-modulated (FM) arm that arises from changes in the firing frequency of sympathetic, parasympathetic, and sensory nerves that innervate bone. Here, we review the medical consequences arising from the dysfunction of the AM and FM arms, as well as studies that have unmasked promising therapeutic targets.

## Introduction

The past decade has witnessed breakthroughs in our understanding of the molecular underpinnings of bone remodeling — a process that continues throughout adult life — in which packets of old bone are replaced by packets of new bone to maintain skeletal integrity (1). Its disruption underscores the pathogenesis of a number of bone and mineral diseases, including osteoporosis, which affects approximately 200 million people worldwide, and in which bone resorption by osteoclasts exceeds bone formation by osteoblasts, leading to net bone loss and a high risk of fracture. Disrupted bone remodeling also underpins the skeletal fragility in bone metastasis, inflammatory joint and tooth diseases, hyperparathyroidism, and rare genetic bone disorders.

Studies on hypothalamic leptin and its downstream relay first established that sympathetic outflow negatively regulates bone formation (2–4); this we term “frequency modulation” (or FM) (4). Subsequent studies from our lab and others revealed that neurohormones from the pituitary gland can act directly on bone cells (4–6). This form of “amplitude modulation” (AM) is exerted by thyroid-stimulating hormone (TSH), follicle-stimulating hormone (FSH), adrenocorticotropic hormone (ACTH), prolactin, oxytocin (OXT), and arginine vasopressin (AVP) (5–12). In this framework, FM refers to changes in the firing frequency of nerves innervating bone, whereas the AM arm is exerted through alterations in circulating hormone concentrations. These studies also provided the first glimpse into the ubiquity of pituitary hormone action in line with the highly distributed functions of ancient precursors, contrary to the prevailing view that these hormones have singular functions in physiology (13–15).

We now have granular detail on the neural innervation of bone, as well as the connectivity of bone to central neurons via sympathetic fibers (16). These explorations have been made possible by the use of pseudorabies virus strains, such as PRV152, that ascend retrogradely from the periphery to the CNS by traveling along sympathetic nerves (16, 17). It has also been gleaned that there is retrograde functional connectivity between the brain and bone, with the latter now widely considered an endocrine organ. For example, it has been suggested that the bone protein osteocalcin may affect behavior and cognition through its action on neuronal GPR158 receptors. Studies have also identified G protein–coupled receptors for pituitary hormones in the brain itself, suggesting their role in the central regulation of multiple bodily processes through somatic innervation (18, 19). Here, we review emerging mechanisms through which the skeleton is regulated by neurohormones and nerves. We also provide new explanations for clinical consequences of disrupted mechanisms, as well as novel translational insights.

## Neuroendocrine regulation of bone mass — the AM arm

### FSH directly causes bone loss.

A direct effect of FSH on bone, independently of estrogen, was first proposed by the demonstration that haploinsufficient *Fshb*^+/–^ mice display a high bone mass in the setting of unaltered ovarian function (6). Since then, multiple studies have shown that in mice, FSH exacerbates bone loss induced by ovariectomy, and conversely, FSH antagonists, including our FSH-blocking antibody, are protective in that context (20–24). Likewise, rats injected with the ovotoxin 4-vinylcyclohexene diepoxide, which causes protracted ovarian failure mimicking human perimenopause, lose approximately 10% of their skeleton during the high-FSH/normal-estrogen phase, thus implicating FSH as an independent driver of bone loss in this model (25).

That FSH receptors (FSHRs) are present on bone cells, namely osteoclasts and osteoblast precursors, has been widely confirmed across species (26). FSH promotes osteoclast formation directly through an FSHR isoform lacking exon 9 and, by interacting with a Gi, reduces cAMP levels to stimulate MAP kinase and IκB pathways (6, 27–29). Unlike the canonical ovarian FSHR that is coupled to Gs, the Gi-coupled FSHR isoform is also expressed in adipocytes, where its activation decreases cAMP and UCP1 expression (24). FSH also promotes osteoclast formation indirectly by augmenting inflammatory signals, including RANK, IL-1β, TNF-α, and IL-6 (30–32). However, FSH-induced osteoclastogenesis does not occur in the absence of immunoreceptor tyrosine–based activation motif (ITAM) adapter signaling molecules (33).

**Figure 1:**
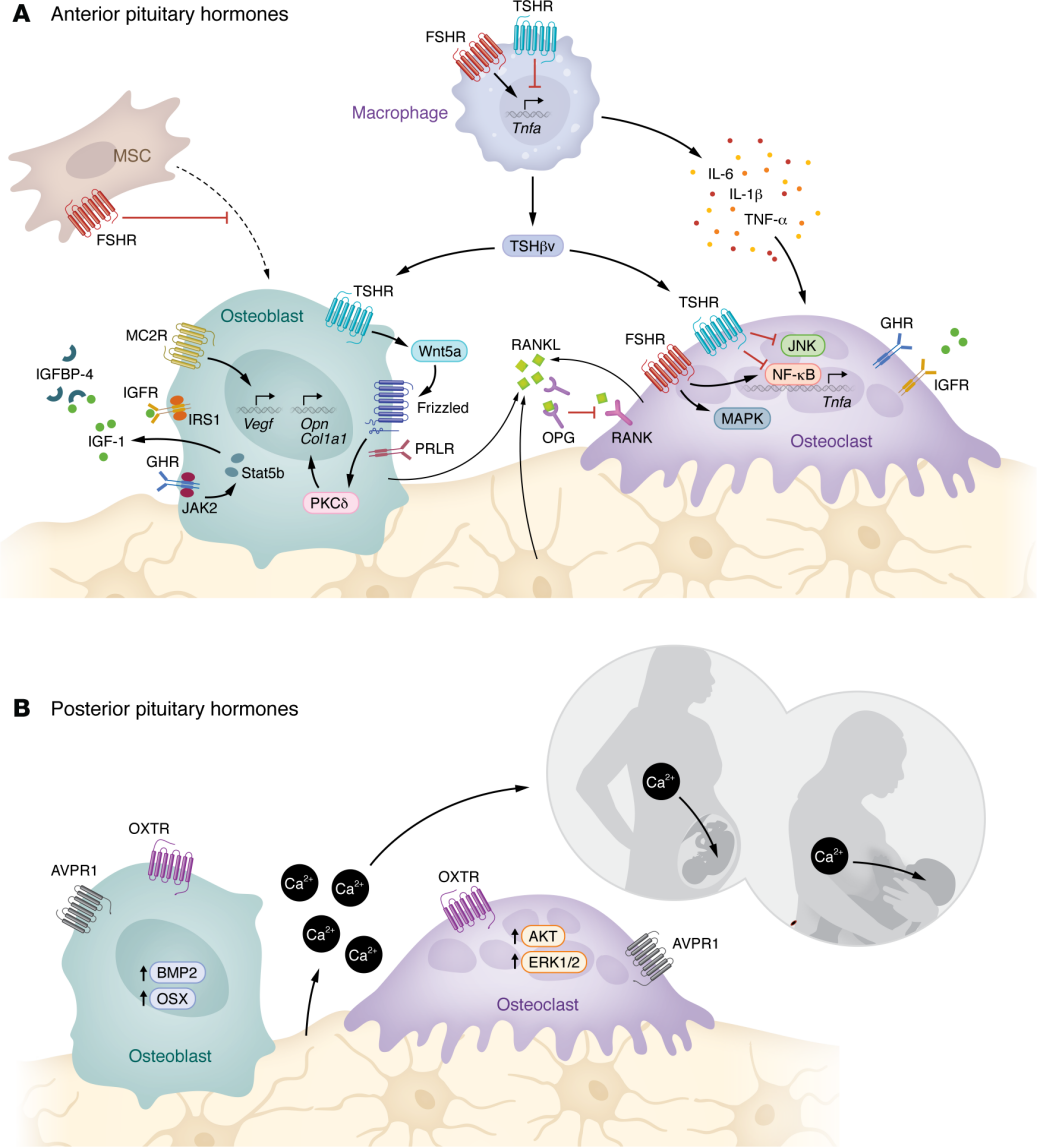
Pituitary hormones directly regulate bone cells — the AM arm. (**A**) Anterior pituitary hormones act on both osteoblasts and osteoclasts via G protein–coupled receptors and, indirectly, through cytokines. FSH interacts with FSHRs on osteoclasts and on macrophages; the latter release TNF-α, which, in turn, promotes bone resorption. FSH also acts on FSHRs on osteoblast precursors to suppress their differentiation. TSH promotes bone formation via Wnt5a, while inhibiting osteoclastic bone resorption by downregulating the NF-κB pathway. A TSH variant, TSHβv, released from immune cells also acts on the same TSHRs on bone cells. Osteoblastic MC2R activation by ACTH upregulates VEGF. GH and IGF-1 promote bone remodeling via JAK2/STAT5b and IRS-1, respectively. Locally derived IGFBP scavenges IGF-1. (**B**) The posterior pituitary nonapeptide oxytocin (OXT) increases bone turnover by acting on osteoblast OXTRs to promote bone formation through BMP2 and osterix (OSX) upregulation. Osteoclastogenesis is also enhanced via the upregulation of AKT and ERK1/2 signaling, which, in turn, facilitates maternal-fetal calcium transfer during pregnancy when serum OXT levels are high. Arginine vasopressin receptor 1a (AVPR1a) opposes OXT action to inhibit bone formation. MSC, mesenchymal stem cell; MC2R, melanocortin 2 receptor; IRS, insulin receptor substrate; IGFBP, insulin growth factor–binding protein; OPG, osteoprotegerin; Opn, osteopontin; Col1a1, collagen 1α1.

In addition to enhancing the formation, migration, function, and survival of osteoclasts (28, 34), FSH suppresses osteoblast differentiation. Accordingly, blocking FSH action with our anti-FSH antibody promotes osteoblast precursor differentiation and upregulates the osteoblastogenic gene program, resulting in increased new bone formation (21, 23, 27, 35). Thus, the attenuation of FSH action not only decreases osteoclastic bone resorption, but also, in parallel, enhances osteoblastic bone formation (Figure 1). Given that the rapid bone loss in perimenopausal women is likely driven, at least in part, by rising FSH levels, circulating FSH is now considered a promising target for mitigating bone loss.

It is notable that the rapid bone loss during the perimenopausal transition occurs around three years before the final menstrual period, when FSH levels start rising but estrogen is relatively unperturbed (36, 37). Multiple observational studies, including large cohorts of individuals of diverse ethnicities and ages, have documented strong associations between high serum FSH levels and bone loss. The Study of Women’s Health Across the Nation (SWAN), a longitudinal cohort of 2,375 perimenopausal women (42 to 52 years old), provided the clearest evidence, showing not only that a point estimate of high FSH correlates with a point estimate of low bone mineral density (BMD), but that the extent to which serum FSH rises over 4 years predicts the magnitude of decline in BMD (37). These observations suggested, for the first time, that serum FSH may be a better predictor of BMD loss than estrogen, at least in the perimenopausal phase.

The National Health and Nutrition Examination Survey (NHANES) III and other large observational cohorts independently report associations between high FSH levels and increases in markers of bone resorption or low BMD (38–41). The Bone Turnover Range of Normality (BONTURNO) study showed that women with serum FSH levels greater than 30 IU/L have higher bone turnover markers than age-matched women with FSH below 30 IU/L (42). Unlike SWAN, BONTURO further suggested that the association between serum FSH and bone loss is not limited to the perimenopausal period but extends into postmenopause. Indeed, analysis of an even older cohort, the Age, Gene/Environment Susceptibility–Reykjavik (AGES-Reykjavik) study of older adults from Iceland, comprising individuals of a mean age of 76 to 79 years, revealed a positive correlation between serum FSH, bone turnover and, importantly, a high risk of incident hip fracture (43, 44).

The inverse correlation between serum FSH levels and BMD has also been noted in patients with Turner syndrome, wherein ovarian insufficiency causes hypergonadotropic hypogonadism (45). Notably, PBMCs cultured from patients with Turner syndrome with high FSH levels showed upregulated osteoclastogenesis, with a higher expression of RANK and TNF-α, suggesting, once again, that FSH causes bone loss by promoting osteoclastic bone resorption in humans (46). Genetic evidence in postmenopausal women also supports a role for FSH in human bone loss. The genotyping of 289 unrelated postmenopausal women revealed that rs6166, an activating polymorphism within the coding region of the *FSHR* gene, was associated with high bone resorption and low bone mass (47). Finally, the GnRH agonist luprolide, which lowers serum FSH levels, has been shown to increase procollagen type I N-terminal propeptide, a bone formation marker, as well as bone resorption markers (48). This high turnover state may result from the coupling of resorption to elevated bone formation induced by suppressed FSH, although other hormonal perturbations from luprolide itself could confound interpretation.

### Targeting FSH for osteoporosis and aging disorders.

Although beyond the scope of this Review, FSH also stimulates adipogenesis, and acts on FSHRs in the dentate gyrus of the hippocampus to provoke neurodegeneration and memory loss in mice (24, 49, 50). Blocking FSH action, genetically or pharmacologically, prevents these effects (6, 21, 23, 24, 35, 49, 51, 52). Notably, osteoporosis, obesity, and Alzheimer disease track together from the beginning of late perimenopause and beyond (53–55). Thus, with the aim of suppressing the pathological actions of FSH, we developed an array of FSH-blocking antibodies for potential future use in osteoporosis, obesity, and Alzheimer disease. The antibodies were raised against a computationally defined 13-mer epitope on FSHβ, binding to which blocks the interaction of FSH with its receptor. We find that these polyclonal and monoclonal antibodies prevent post-ovariectomy bone loss, diet- and ovariectomy-induced obesity, and ovariectomy-induced neurodegeneration and memory loss in mice (21, 23, 24, 49).

Subsequently, we humanized our murine monoclonal FSH-blocking antibody. Our Investigational New Drug–enabling studies show that the lead candidate, MS-Hu6, and its murine version, Hf2, are stable and adequately bioavailable; display extended pharmacokinetics, FSH engagement, and efficacy in mouse models; and are safe in monkeys (35, 52, 56–58). Specifically, in the context of osteoporosis, we find that MS-Hu6 not only prevents bone loss but also rescues established osteoporosis (52). We have also obtained the crystal structure of the Fab fragment of MS-Hu6 (56), and the antibody is now undergoing good manufacturing practice development along with toxicology studies toward a first-in-human trial. In addition to our antibody, other approaches, such as vaccines and aptamers, are also being tested to target FSH for osteoporosis and other aging disorders (50, 51) (Table 1).

### Low TSH exacerbates bone loss from hyperthyroidism.

The finding that mice haploinsufficient for the TSH receptor (*Tshr*^+/–^) displayed osteopenia in the face of normal thyroid development and function was the first evidence that a pituitary hormone could act on a somatic tissue by bypassing its traditional target — in this case, the thyroid gland (5). Moreover, homozygous *Tshr*^–/–^ mice, even when given thyroid hormone replacement, displayed low bone mass, and these mice lost more bone than wild-type littermates when T4 pellets were implanted (59). This reduction in bone mass results from increased osteoclast formation, in large part due to elevated TNF-α expression (6, 30). Treating *Tshr*^–/–^ mice with an anti–TNF-α neutralizing antibody reverses the increased osteoclastogenesis (60). Similarly, deletion of TNF-α from the *Tshr*-deficient background results in gene-dose-dependent rescue of the low bone mass phenotype in the compound mutants (61). In addition to being an antiresorptive hormone, TSH also promotes bone formation. While in vitro data with cell lines initially indicated the inhibition of osteoblastogenesis, intermittently injected TSH was found to increase bone formation in both wild-type and ovariectomized mice (9, 62). In embryonic stem cell–derived osteoblasts, TSH activates PKCδ and upregulates several anabolic signals, including the noncanonical WNT components FRZ and WNT5a (63) (Figure 1).

Not only does TSH act on bone cells, its splice variant, TSHβv, is expressed in bone marrow in CD11b^+^ macrophages and other immune cells (64). Bone marrow levels of TSHβv are not regulated reciprocally by thyroid hormones, explaining how local TSH may be bone protective, despite low pituitary-derived circulating TSH in hyperthyroidism (64, 65). It is therefore expected that activating TSHR antibodies, for example those detected in autoimmune thyroid disease, may protect against severe hyperthyroid bone loss. We have shown that in vitro, a TSHR-agonist antibody reduces osteoclast formation and the osteoclastogenesis gene program (66).

Hyperthyroidism-associated bone loss is linked to an increased risk of fracture (67). While it is clear that thyroid hormone stimulates bone resorption through thyroid receptor α1 (68–70), there is considerable evidence from human studies that TSH has an independent action on bone mass regulation. Studies using multiple cohorts of euthyroid individuals, as well as patients with subclinical hyperthyroidism with low TSH and normal T3/T4 levels, show that low serum TSH itself associates with low BMD and increased fracture risk (71). Notably, the Study of Osteoporotic Fracture (SOF) showed that postmenopausal women with low TSH (<0.1 mIU/L) had a higher risk of hip, vertebral, and nonvertebral fracture compared with those with normal TSH levels (0.5–5.5 mIU/L) (72). Furthermore, in studies in patients with thyroid cancer undergoing TSH suppression, TSH levels were inversely correlated with the risk of radiographic vertebral fracture, independent of age, BMD, and thyroid hormone levels (73, 74). However, patients with subclinical hypothyroidism did not show any marked difference in BMD or fracture risk compared to euthyroid individuals (75, 76). TSH-secreting pituitary adenomas, however, are associated with an increase in bone resorption and morphometric vertebral fractures, likely due to elevated T3/4 levels (77). Genetic data, albeit limited, show that a SNP in the coding region of *TSHR*, Glu727, is associated with osteoporosis (78). Several interventional studies in humans further show that recombinant human TSH (rhTSH) affects bone turnover markers. For example, patients injected with rhTSH showed increased bone formation markers, namely bone alkaline phosphatase and P1NP, and decreased bone resorption markers, such as serum C-telopeptide and urinary N-telopeptide (79–81). In all, whereas the TSHR is unlikely to become an actionable target for osteoporosis, these studies are instructive in that they alert endocrinologists not to oversuppress TSH levels in patients on thyroid hormone therapy, unless it is necessary to do so in those with thyroid cancer.

### Consequences of anabolic action of ACTH on bone.

The ACTH receptor, MC2R, is expressed on both osteoblasts and osteoclasts (82, 83), and ACTH displays direct effects on bone remodeling (12). In patients with adrenal Cushing syndrome with suppressed ACTH have lower BMD than those with pituitary Cushing disease with elevated ACTH (84), suggestive of independent skeletal protection by ACTH. Conceptually, therefore, suppressed ACTH, which is often noted in the setting of chronic glucocorticoid use, may contribute to glucocorticoid-induced bone loss, although this hypothesis requires experimental testing. However, a protective effect of ACTH has been observed in a rabbit model of glucocorticoid-induced osteonecrosis. Injected ACTH attenuates glucocorticoid-induced osteonecrosis and upregulates the osteoblastogenesis gene program, as well as *Vegf* and *Tgfb* expression (12) via α-2-macroglobulin (85, 86) (Figure 1). However, contrary to the anabolic effect of ACTH signaling, *Mc2r*^−/−^ mice exhibit increased bone formation and cortical bone mass. These mice, nonetheless, also have adrenal insufficiency, a potential confounder (87). Further studies using osteoblast-specific *Mc2r*-null mice, without the confounding effect of low cortisol levels, should provide better insight into the putative skeletal actions of ACTH.

### Growth hormone action through IGF-1.

Bone is a major target for growth hormone (GH) both in children and adults (88, 89). GH action on bone is mediated primarily through systemic IGF-1 secreted by the liver under GH control (Figure 1). That overexpression of IGF-1 rescues the growth retardation and osteoporosis in GH receptor–deficient (GHR-deficient) mice establishes its downstream anabolic function (90). Consistent with this, and despite elevated GH levels, mice lacking both liver IGF-1 and the acid labile subunit (LID+ALSKO mice), display reduced bone strength (91). The induction of osteoclastic activity by GH also appears to require osteoblast-derived IGF-1, which then activates bone resorption by acting on osteoclast receptors (92, 93). Thus, GH is customarily regarded as a bone-remodeling stimulant that acts via IGF-1 (94). That said, there is also evidence for direct effects of GH on the GHR (88). Notably, GH reverses ovariectomy-induced bone loss in LID mice (95), and also increases adiposity in hypophysectomized rats, whereas IGF-1 replacement does not (96).

### Skeletal effects of GH excess in acromegaly.

Acromegaly is generally caused by a pituitary adenoma secreting GH, which, in turn, leads to increased circulating levels of IGF-I (97). Notwithstanding multiple comorbidities (98), skeletal fragility has emerged as a major clinical consequence of acromegaly (99). High GH causes high bone turnover, with increased bone resorption (100, 101), resulting in high morphometric and incident vertebral fracture (102–105), as well as hip fractures (106). Whereas incident vertebral fractures can occur early in the natural history of the disease, often at diagnosis (107), morphometric fractures are related mainly to active disease duration, coexisting diabetes (108), and serum IGF-I levels (109), but not BMD. In fact, there is an infrequent reduction in lumbar spine BMD (100), and fractures may occur in patients with normal or minimally decreased BMD (102). Expectedly, GH-lowering agents reduce fracture risk (110), with the second-generation somatostatin analog pasireotide being more efficacious than the GH antagonist pegvisomant (111), suggesting a possible bone-protective effect of the somatostatin analog per se.

It follows that, in patients with acromegaly, trabecular bone score (TBS), high-resolution peripheral quantitative computed tomography, cone beam computed tomography, and bone microindentation are more reliable predictors of fracture risk than BMD (112). The most recent consensus guidelines recommend assessment of BMD and vertebral morphometry at the diagnosis of acromegaly (113). Skeletal monitoring should be standard-of-care at pituitary tumor centers of excellence (114). In fact, combined measures of biochemical control (IGF-1) and of bone quality, such as TBS, may allow a better prediction of fracture risk, and guide early and effective skeletal protective strategies at diagnosis and follow-up (112).

### Skeletal fragility in GH deficiency.

GH deficiency (GHD) in adults in the context of anterior hypopituitarism arises most commonly from pituitary surgery or empty sella (115, 116). Among associated comorbidities (117), there is an increased fracture risk due to a low turnover state with reduced bone formation and resorption (118–120). BMD can be low or normal (118). Patients with childhood-onset GHD display a more severe phenotype, with other modifiers being disease severity (GH peak <3 ng/mL after GHRH plus arginine) (118), glucocorticoid over-replacement for secondary hypoadrenalism (121), and TSH deficiency (see above). While data on bone quality at baseline or after GH replacement in adult GHD are scant, limited studies suggest that post-replacement bone quality, measured with TBS, is neither altered markedly (122) nor does it improve, except in vitamin D–deficient patients (123).

Patients with GHD and individuals with a loss-of-function *GHR* polymorphism have an increased risk of morphometric vertebral fractures (124–126). In untreated patients, fracture prevalence is related to low BMD and disease duration (124), and new vertebral fracture incidence tracks with prevalent vertebral fractures and low lumbar BMD (125). GH replacement can lower fracture risk without altering BMD, depending on the time of therapeutic initiation (124). Skeletal remodeling is restored through an early rise in bone resorption followed by increases in bone formation at 9 to 12 months. This sequence is consistent with the initial decline in BMD noted during the first year of high-dose treatment for adult-onset GHD, with GH-driven improvements in BMD emerging typically only after 18 to 24 months of replacement (120). There is often a blunted or delayed response in patients with preexisting Cushing disease or prior acromegaly (127). Accordingly, BMD measurement and vertebral morphometry are standard-of-care at the time of diagnosis and in the long term.

### Oxytocin and osteoporosis of pregnancy and lactation.

During pregnancy and lactation, the mother’s skeleton adapts to meet the needs of fetal and postnatal skeletal mineralization, resulting in increased maternal bone resorption and intergenerational calcium transfer (128, 129). Remarkably, this skeletal loss is restored through increased maternal bone formation when the requirement for resorption recedes during weaning. However, the risk of low bone mass and even vertebral fracture remains high and can result in a distinct clinical entity known as osteoporosis of pregnancy and lactation (128, 130). In addition to hypogonadism per se and placenta-derived parathyroid hormone–related peptide (PTHrP) (131), OXT, which rises during pregnancy and lactation, appears to have a role in maternal bone mass regulation. The role of prolactin is poorly understood, except that prolactin receptor–deficient *Prlr*^–/–^ mice, and paradoxically, patients with hyperprolactinemia and prolactinomas, display reduced BMD and increased risk of vertebral fractures (132–135) (Figure 1).

OXT receptors (OXTRs) are present on both osteoblasts and osteoclasts (10, 136). *Oxt*^–/–^ and *Oxtr*^–/–^ mice display profound age-dependent bone loss, with reduced bone formation (10). OXT replacement reverses postovariectomy bone loss and stimulates new bone formation by acting on osteoblast OXTRs — an effect that is mediated, in part, by OXTR nuclear translocation (10, 137, 138). Osteoblast-specific *Oxtr* mutants not only display osteopenia, but are protected from bone loss during pregnancy and lactation, likely through the inhibition of osteoclasts (7). This is consistent with the prevention by OXT of resorption by active osteoclasts, while it stimulates osteoclastogenesis (7, 10). Accordingly, osteoclast-specific *Oxtr* mutants display high bone mass, supporting a role for OXT in early skeletal loss from enhanced osteoclast formation. In concordance, *Oxt*^–/–^ fetuses show reduced trabecular mineralization (7, 139, 140). Collectively, therefore, while high OXT levels induce intergenerational calcium transfer by mobilizing calcium from the maternal skeleton, they also induce new bone formation to restore the lost skeleton during weaning (141).

### Water balance and bone loss.

Chronic hyponatremia is associated with osteoporosis and a high fracture risk (142–144), toward which the role of elevated arginine vasopressin (AVP), a primary regulator of serum osmolality and fluid balance, has assumed importance. As OXT and AVP share a common ancestral precursor, mesotocin, the advent of distributed functions of the two nonapeptides in mammals may suggest opposing actions, which is the case with bone (Figure 1). Thus, in contrast with *Oxtr* deficiency, mice lacking AVP receptor 1a (*Avpr1a^−/−^*) show increased bone formation and decreased resorption, with net bone gain (8, 11). This high bone mass phenotype is rescued in *Oxtr^−/−^*;*Avpr1a^−/−^* double mutants. Pharmacologic intervention with AVP or AVPR1a antagonist reduces or increases bone mass, respectively (11), while blocking AVPR2 in the kidney by tolvaptan spares the skeleton (8). In all, given that bone is the largest reservoir for sodium, high AVP levels in patients with chronic hyponatremia may signal to trigger bone resorption for sodium release into the circulation (145, 146).

## Neural regulation of bone remodeling — the FM arm

### Neural innervation of bone.

Neuroanatomical evidence for sympathetic nervous system (SNS) innervation of lower limb bones has come from viral transneuronal tract tracing using the pseudorabies virus strain PRV152 (147, 148), which travels retrogradely from postganglionic neurons (mostly L1–L2) to SNS preganglionic neurons in the intermediolateral (IML) column of the spinal cord and, finally, ascends to the brain, thus identifying the entire bone (femur)-brain neuroaxis (16). Following injection of virus into bone tissue, PRV152-infected SNS neurons are detected in the hypothalamus, particularly in the paraventricular hypothalamic nucleus (PVH), lateral hypothalamus (LH), and dorsomedial hypothalamus (DMH), as well as in the medulla, midbrain, pons, and forebrain (16). Likewise, a hierarchical circuit controlling SNS output to bone marrow has been identified by detecting PRV152 in ganglia and the paravertebral chain in the IML of the lower thoracic spinal cord (149). Neurons in C1, A5, and A7 catecholaminergic cell groups, other nuclei of the ventrolateral and ventromedial medulla, the periaqueductal gray, PVH, and other hypothalamic nuclei, as well as the insular and piriform cortices constitute the central network for SNS outflow to bone marrow (149). These data suggest that the SNS neural circuitry that innervates bone marrow also connects the brain to bone.

The β_1_- and β_2_-adrenergic receptors are expressed in human and murine bone. The best human evidence for a direct role of the SNS in bone remodeling comes from patients with sympathetic osteodystrophy, which is associated with severe local bone loss (150). Patients with pheochromocytoma with increased SNS tone also present with high bone turnover, reduced bone mass, impaired bone quality, and an increased fracture risk (151, 152). Interestingly, while clinical trials show that β_1_-selective blockers increase BMD at the distal radius and improve microarchitecture (153), observational studies remain inconsistent (154–156). Finally, in developmental models, denervation by sciatic neurectomy or sympathectomy results in impaired longitudinal growth and arrested bone mass accrual (157), as well as increased osteoclasts on the bone surface (158).

Opposing the effects of SNS relay, central parasympathetic control of bone occurs through muscarinic receptors (M3Rs) in noradrenergic neurons of the locus coeruleus (159). Global and neuron-specific *M3r*-deficient mice thus display low bone mass (159). In the periphery, parasympathetic nerve terminals originating from the spinal cord release acetylcholine (ACh) to interact with nicotinic ACh receptors (nAChRs) present predominantly on osteoclasts. nAChR antagonists cause osteoclast apoptosis and reduce bone resorption, whereas mice deficient in the α2-nAChR subunit display elevated bone resorption and low bone mass (160). In addition, mice with reduced activity of the choline transporter ChT with less ACh synthesis are osteopenic (161). Interestingly, cytokines such as IL-6 cause a cholinergic switch of sympathetic noradrenergic neurons, which is mediated through an interaction of the GDNF family receptor GFRα2 with its ligand, neurturin, to promote survival and connectivity of bone-embedded osteocytes (162).

Peripheral sensory axons also innervate the periosteal layer of bone and bone marrow, with different densities and distribution patterns (163). In patients with hyperparathyroidism or a new fracture, the innervation is denser and is located above the remodeling surface (164, 165), while overall innervation density decreases with aging (166) and in diabetic neuropathy (167). Multiple molecular pathways are also used to transmit sensory signals from the skeleton. Deletion of the axonal factor semaphorin-3A (168) globally or in neurons yields a low bone mass phenotype (169). Likewise, nerve growth factor governs the innervation of the developing femur and supports vascularization and osteogenesis via the neurotropic tyrosine kinase receptor type 1 (170).

Of the sensory neuropeptides found in bone, substance P and αCGRP are the most well investigated. Substance P stimulates osteoblast and osteoclast differentiation and enhances bone regeneration during fracture healing through the neurokinin-1 receptor (171–173). In contrast, while αCGRP upregulates osteoblast differentiation (174), it inhibits osteoclastogenesis and bone resorption (174–176). Global deletion of the *Calca* gene, which encodes both calcitonin and αCGRP, surprisingly yields a high bone mass phenotype, suggesting an independent skeletal action of αCGRP (177, 178). Indeed, αCGRP-deficient mice exhibit decreased bone formation and trabecular bone mass with no change in cortical bone (177). However, these mice also display decreased αCGRP reactivity in dorsal root ganglia and a loss of αCGRP-positive nerve fibers in bone (177), indicating that some of the phenotypic effects might be of neural origin. Denervation by capsaicin to reduce substance P or αCGRP in nerves likewise results in impaired bone formation and bone loss (179, 180). In contrast, increasing αCGRP expression in lumbar dorsal root ganglia and αCGRP-positive sensory innervation in the periosteum by continuous magnesium infusion accelerates cortical bone formation during fracture healing.

αCGRP- and substance P–mediated sensory signaling thus represents an integral regulatory component of bone remodeling and bone healing, rather than a parallel consequence of nociceptive activation. The two neuropeptides are frequently coexpressed in the same primary afferent sensory neurons, and tissue injury triggers their coordinated co-release (181). During fracture repair and bone regeneration, primary sensory and SNS fibers rearrange and reinnervate the site of bone injury (182, 183). A critical mediator of this crosstalk during fracture repair is neural FGF9 (184). Genetically modifying *Fgf9* expression in dorsal root ganglia and sensory neurons promotes bone regeneration (184). These findings imply that nociceptor activation and neurogenic inflammation initiate neural-bone crosstalk to shape the local microenvironment in favor of bone repair. Nevertheless, the mechanisms by which nociceptive sensory neurons undergo a functional transition from being nociceptive to a trophic, growth factor–secreting phenotype, remain poorly understood.

In all, there is clear evidence for a functional interaction between bone and its innervation by both SNS and sensory nerves, with the latter limited to first order sensory neurons that project rostrally as a sensory inflow pathway to the brain from bone. An intriguing possibility, which requires further investigation, is that bone catabolic effects, triggered by increased SNS inflow, are sensed and counterbalanced by sensory outflow.

### Hypothalamic circuitry in bone remodeling.

**Figure 2:**
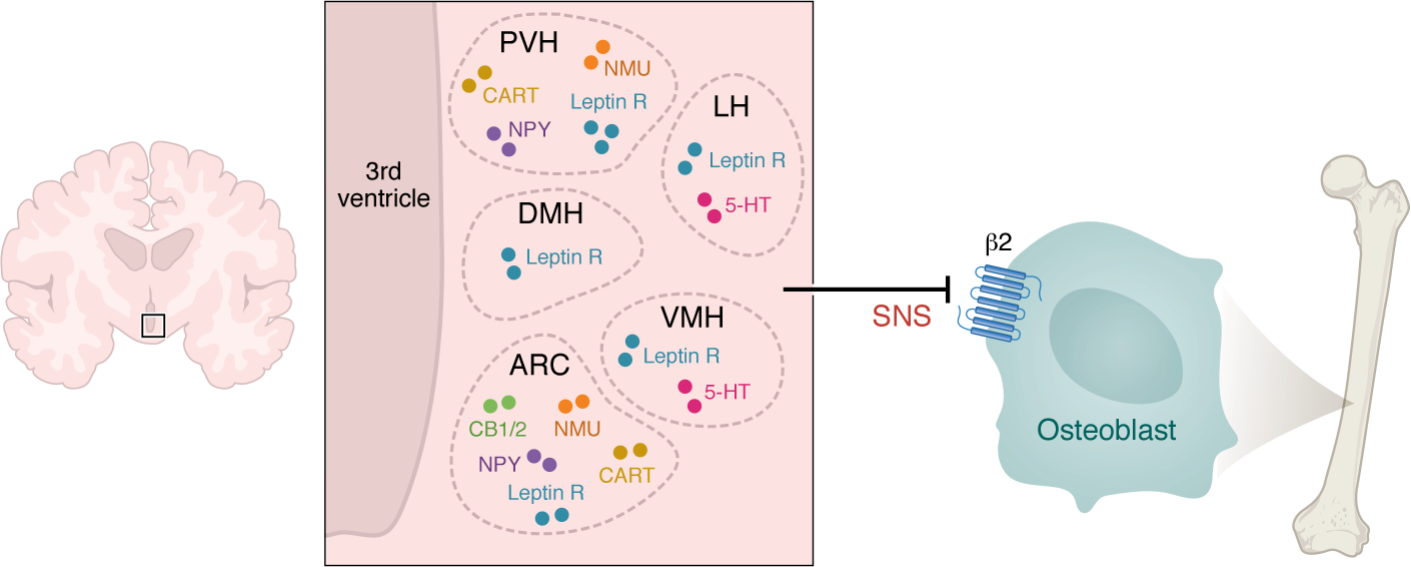
Central neural regulation of bone remodeling — the FM arm. Neural circuits originating from the hypothalamus, namely CART, CB1/2, NPY, NMU, and 5-hyroxytryptamine (5-HT), affect bone remodeling primarily by altering sympathetic tone, an effect that is, in part, mediated through central leptin. Hypothalamic regions, such as the arcuate nucleus (ARC), paraventricular hypothalamic nucleus (PVH), dorsomedial hypothalamus (DMH), lateral hypothalamus (LH), and ventromedial hypothalamus (VMH) are critical to bone mass regulation, but also act as central nodes for controlling feeding behavior and energy balance.

Leptin receptors are present in the arcuate nucleus (ARC) and other hypothalamic areas such as the ventromedial hypothalamus (VMH), DMH, and LH, to regulate appetite and energy expenditure (185). Intracerebroventricular (ICV) injection of leptin in leptin-deficient *ob/ob* mice attenuates the high bone mass (2). This is consistent with the low bone mass observed in gain-of-function leptin receptor mutants (3), and the high bone mass seen in leptin receptor–deficient (*db/db*) mice (3). Importantly, ICV leptin fails to reduce bone mass in mice deficient in dopamine β-hydroxylase (DBH) (3, 186), providing clear evidence that the SNS relay to bone is downstream of hypothalamic leptin signaling. Likewise, ICV leptin fails to reduce bone mass in mice lacking β_2_-adrenergic receptors specifically in osteoblasts (187). Mechanistically, leptin-induced sympathetic outflow is initiated by the transcription factor FOXO1, which upregulates DBH expression in SNS ganglia (187). Activation of ADRB2, in turn, stimulates the expression of molecular clock genes, including *Per* and *Cry*, which increase bone remodeling by inducing *cFos* and *Jun* expression (188). These studies firmly point to a role for the SNS in regulating bone mass, and account for the central action of leptin (Figure 2).

Apart from leptin receptors, neurons of the PVH and ARC express cocaine- and amphetamine-regulated transcript (CART), neuropeptide Y (NPY), and neuromedin U (NMU). Beyond their roles in regulating eating behaviors and energy balance, these neuropeptides also control bone remodeling. CART is upregulated by leptin and is not expressed when leptin is absent in *ob/ob* mice (189, 190). *Cart*^–/–^ mice display low bone mass, with a further decease in bone mass upon ICV leptin injection (190), indicating that leptin-mediated SNS regulation of bone is not dependent on CART (190). In contrast, NMU-deficient mice display high bone mass like *ob*/*ob* mice, but do not respond to ICV leptin (191), suggesting that NMU may, in fact, be required for leptin-induced bone loss via SNS activation.

Leptin receptors also colocalize with NPY receptors in ARC neurons (192, 193), and like ICV leptin, ICV NPY causes bone loss. Consistent with this, global NPY deficiency results in increased osteoblastic activity and bone mass. *Npy2*^–/–^ mice display higher trabecular bone mass (194), while mice selectively lacking *Npy2* receptors in the hypothalamus show high cortical mass (193, 195, 196). However, deletion of NPY2 receptors in NPY-expressing neurons yields only modest increases in cancellous bone and no effect on cortical bone (197). NPY1 receptor deficiency also gives rise to a high bone mass phenotype; however, conditional deletion of hypothalamic NPY1 receptors does not alter bone mass, suggesting that NPY1 may act peripherally, likely through osteoblastic receptors (198).

The ARC and PVN also express cannabinoid receptors, particularly the CB1 receptor. *Cb1*^–/–^ mice display increased bone mass and protection against ovariectomy-induced bone loss, with synthetic CB1 antagonists being osteoprotective (199). Peripherally, CB1 in sympathetic terminals regulates the endocannabinoid 2-arachidonoylglycerol and inhibits norepinephrine release and *Adrb2* activation, thus suppressing enhanced bone turnover (200). CB2 is expressed mainly in bone (201, 202) and an SNP in the *CNR2* gene has been linked to low BMD (203). *Cb2*^–*/*–^ mice thus display accelerated age-related cancellous bone loss resulting from a high turnover state, also marked by increased bone formation (201). While the relevance of these observations to human bone remains unclear, the prevalent use of medical and recreational cannabis underscores the need to evaluate the effects of endocannabinoids on bone health (204).

In contrast, selective serotonin reuptake inhibitor use is associated with reduced BMD and a high fracture risk (205, 206). Mechanistically, central serotonin acts through the HTR2c receptor on serotonergic neurons in the dorsal raphe, which, in turn, communicate with VMH neurons to reduce SNS tone. Thus, CNS-specific *Tph2-*deficient mice display low bone mass (207, 208). There is also a fully functional serotonergic system in osteoblasts and osteocytes, consisting of TPH1, and the 5-HT1A and 5-HT2A receptors (209). Given that gut-specific *Tph1* deletion yields a high bone mass phenotype (207, 208), it has been suggested that serotonin produced from gut enterochromaffin cells suppresses osteoblast proliferation (210), and indeed, blocking TPH1 in the gut prevents bone loss in ovariectomized rodents (211). That said, the role for gut-derived serotonin in mediating the high bone mass phenotype has been challenged (212).

## Hierarchy and convergence of AM and FM control

**Figure 3:**
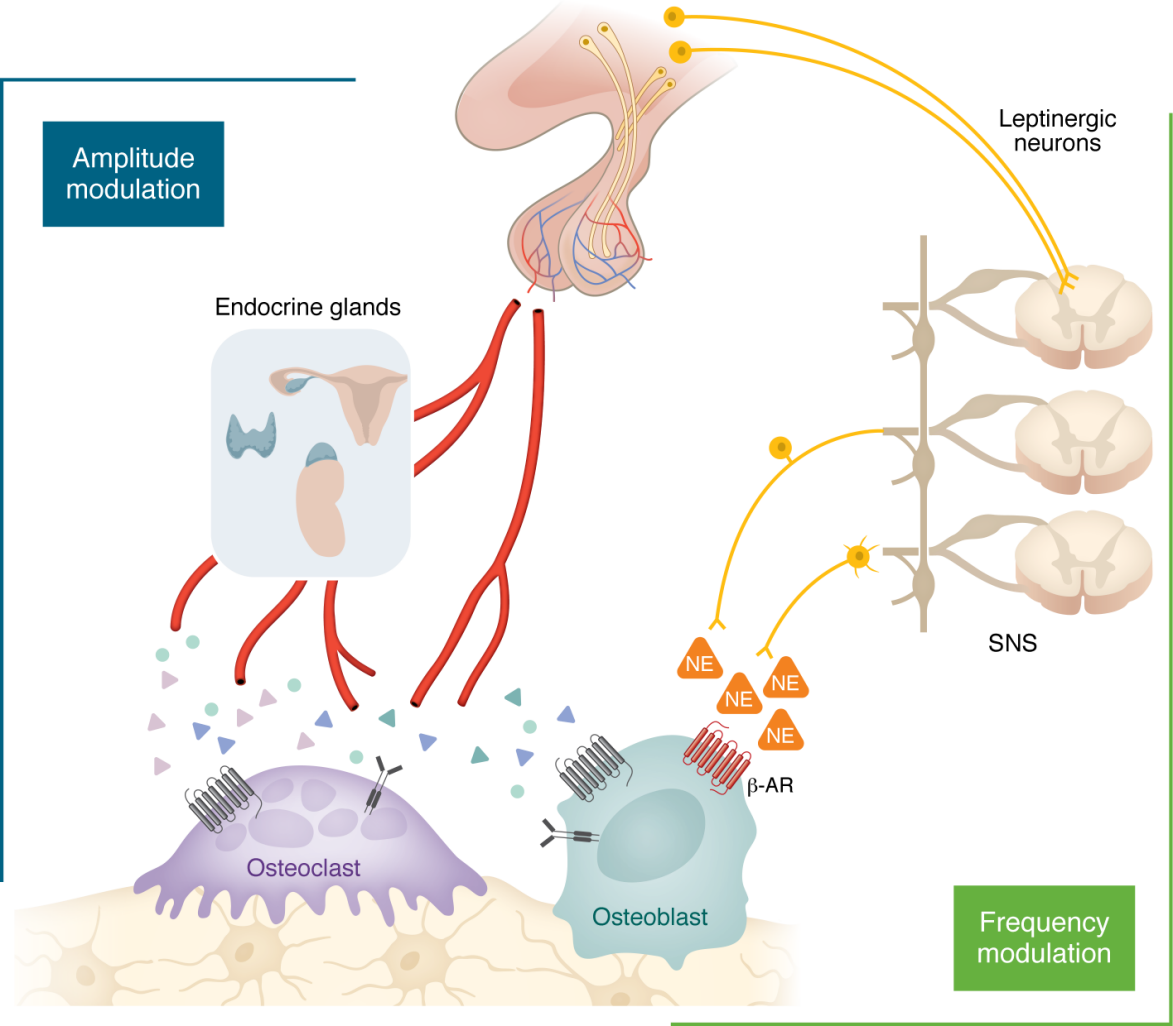
FM and AM arms for central control of bone mass and skeletal integrity. The pituitary gland directly regulates bone remodeling through glycoprotein hormones from the anterior pituitary, as well as hypothalamic nonapeptides released from the posterior pituitary, constituting the AM arm for neural control. In addition, pituitary glycoproteins stimulate the synthesis and secretion of hormones from endocrine glands; the latter have independent actions on the skeleton, which often oppose the direct actions of the glycoproteins themselves. Hypothalamic neurons, under the control of leptin and other hypothalamic neuropeptides, regulate bone remodeling, primarily through changes in the firing frequency of the sympathetic nervous system (SNS), constituting the FM arm. NE, norepinephrine.

Studies collectively suggest that the neural FM and neurohormonal AM arms contribute to maintaining skeletal integrity, serving as buffers to maintain the balance between bone formation and bone resorption when one or the other is perturbed (Figure 3). For example, when FM mode is impaired, such as in *Adr2*^−/−^ mice, ovariectomy, which disturbs the AM arm, does not cause hypogonadal bone loss (190). β-adrenergic blockade likewise increases bone mass not only in normal mice, but also in ovariectomized mice. That β-adrenergic blockade suppresses the otherwise unloading-induced reduction in bone mass, and that β agonists reduce bone mass even despite mechanical loading, suggests that the neural FM arm has a prominent function in the regulatory hierarchy, even overcoming the known effects of mechanical loading.

In normal physiology, AM and FM signals are transient and balanced, and tightly couple osteoclast and osteoblast activities, with pathology emerging mainly when AM signals become chronic, with low or high amplitudes. However, the modes are not dichotomous in that the frequency or periodicity of changes in hormone amplitude matter. Notably, GH secretion is intrinsically pulsatile and circadian, and its physiological effects are shaped by secretion patterns, not merely average concentration. That intermittent TSH injections can generate a potent anabolic effect (9) is one such example. Similarly, while skeletal integrity is unperturbed with periodic FSH surges during cycling, sustained amplification of serum FSH as an adaptive response causes postmenopausal bone loss. Finally, short-lived feeding- and appetite-related hormones, such as GLP-1, GLP-2, GIP, and PYY, regulate bone remodeling directly through the AM arm, as well as indirectly, through the CNS (213, 214). Indeed, we have identified GLP-1–secreting neurons in the nucleus of the solitary tract (215).

## Conclusions

Recent cell-based, mouse genetic, and human studies have revealed anatomical and functional connectivity between bone, neurohormones, and central neurons. Just a few years ago, circulating peptide and steroid hormones, such as parathyroid hormone, 1,25-dihydroxyvitamin D, and sex steroids, among others, were thought to be the sole drivers of bone remodeling. The discovery of neural and neurohormonal networks regulating the skeleton has, however, provided us with several domains of contemplative investigation.

First, and foremost, results from genetically engineered mice lacking ligands and receptors either globally or in bone cells have offered an opportunity to revisit disease pathophysiology (Table 2). Most transformative in this regard has been the idea that postmenopausal osteoporosis may not solely be due to reduced estrogen, but instead, that high FSH levels contribute to bone loss and fracture risk (44). This has been confirmed through the study of multiple patient cohorts across the globe. Second, in-depth anatomical mapping of the neuro-skeletal axis and a clear demonstration of neurohormone receptors in the brain should impel investigators to study an extended range of neural control mechanisms. Third, mouse genetic data, which are consistent with observational, genetic, and interventional studies in people, continue to impact treatment paradigms. Observations that a low TSH level in hyperthyroidism contributes to bone loss have prompted clinicians not to oversuppress serum TSH unless it is necessary to do so in patients with thyroid cancer (71). Likewise, the unexpected bone fragility noted in patients with acromegaly and GHD has prompted intensive skeletal workups for these patient groups (100). Lastly, and equally important, studies on central neural and neuroendocrine control have unmasked new therapeutic targets, such as FSH and its receptor, with wider applications than to osteoporosis alone. New therapeutics, such as our first-in-class FSH-blocking antibody, may soon be developed for treating not only osteoporosis, but also obesity, Alzheimer disease, and other aging disorders that affect millions of women and men worldwide (52, 56).

## Conflict of interest

MZ is an inventor on issued patents on inhibiting FSH for the prevention and treatment of osteoporosis and obesity (US Patents 8,435,948 and 11,034,761). MZ is also an inventor on a patent application on the composition and use of humanized monoclonal anti-FSH antibodies and is a co-inventor on a pending patent on the use of FSH as a target for preventing Alzheimer disease (WO 2023/004044 and WO 2023/004036). MZ and TY are co-inventors on a patent application relating to the ultra-high formulation of an FSH-blocking antibody and on the simultaneous use of the antibody for obesity, osteoporosis, and Alzheimer disease (WO 2024/233558 and US provisional patent 63/831,872). These patents are owned by Icahn School of Medicine at Mount Sinai, and the inventors and co-inventors would be recipients of royalties, per institutional policy. MZ also consults for several financial platforms, including Gerson Lehman Group and Guidepoint, on drugs for osteoporosis and genetic bone diseases. AG participated in Advisory Board meetings for Alexion, Amolyt, and Crinetics Pharmaceuticals and has occasionally consulted for Abiogen, Ipsen, Pfizer, and Recordati and received a research grant to San Raffaele Vita-Salute University from Recordati.

## Funding support

This work is the result of NIH funding, in whole or in part, and is subject to the NIH Public Access Policy. Through acceptance of this federal funding, the NIH has been given a right to make the work publicly available in PubMed Central.

- NIH grant R61 AG094602 (to TY, MZ and VR).
- NIH grant R01 AG071870 (to MZ, TY, and SMK).
- NIH grants R01 AG074092, R01 DK107670, and U0IAG073148 (to TY and MZ).
- NIH grant U19 AG060917 (to MZ and CJR).
- NIH grant RF1 AG093773 (to MZ).

## Figures and Tables

**Table 1:**
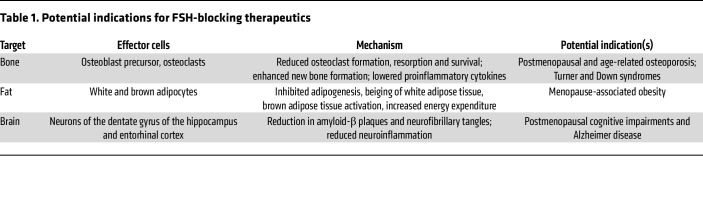
Potential indications for FSH-blocking therapeutics

**Table 2:**
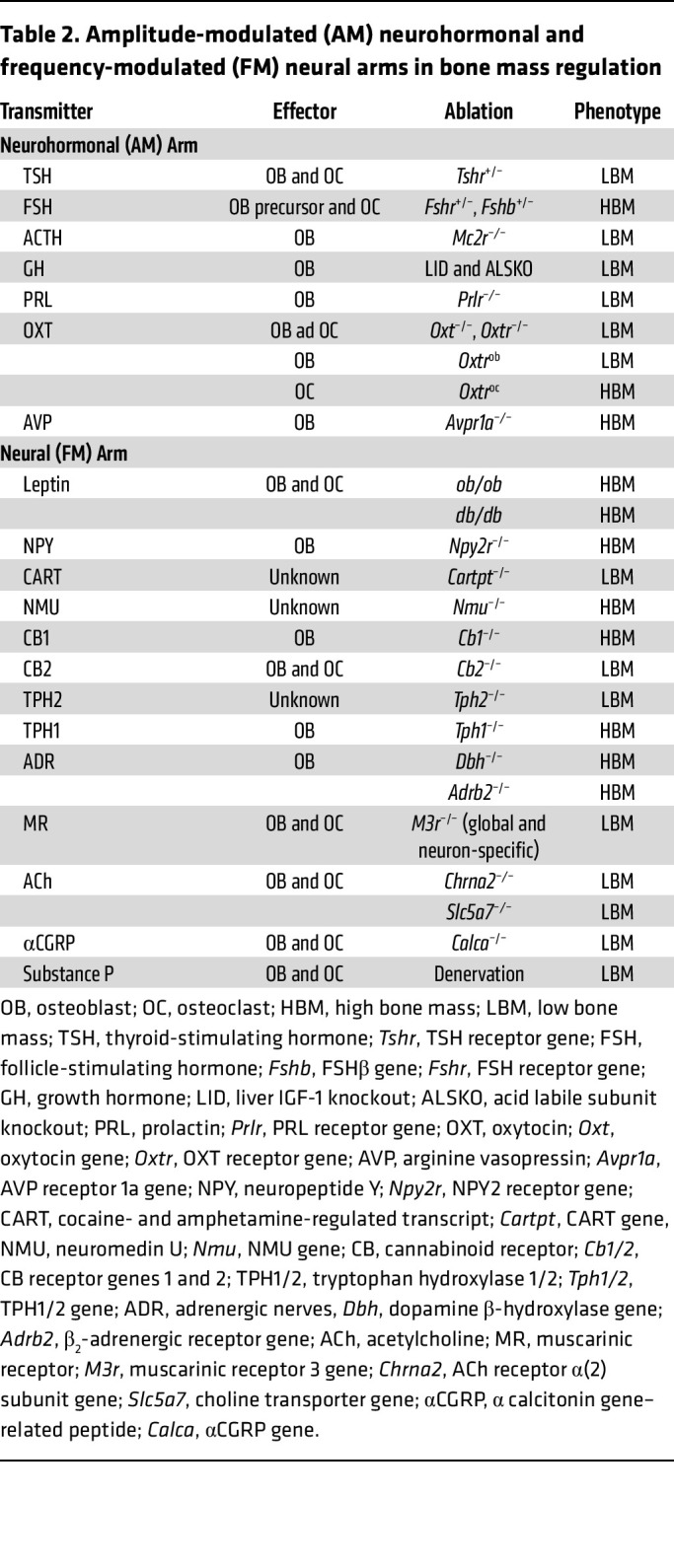
Amplitude-modulated (AM) neurohormonal and frequency-modulated (FM) neural arms in bone mass regulation

## Version 1 05/01/2026

Electronic publication
